# Residual Inflammatory Potential in Patients with Treated Non-Definite and Definite Familial Hypercholesterolaemia: A Clinical Study

**DOI:** 10.3390/jpm16080429

**Published:** 2026-08-14

**Authors:** Patrycja Brzóska-Ritter, Piotr Żarczyński, Maciej Haberka

**Affiliations:** Department of Cardiology, School of Health Sciences, Medical University of Silesia, 40-635 Katowice, Poland

**Keywords:** familial hypercholesterolemia, atherosclerosis, biomarkers, lipoprotein-associated phospholipase A_2_, tumor necrosis factor-α

## Abstract

**Background:** Familial hypercholesterolemia (FH) is associated with accelerated atherosclerosis and an increased risk of cardiovascular disease. **Aims:** To evaluate circulating biomarkers of inflammation and atherosclerosis in patients with clinically suspected FH stratified according to the Dutch Lipid Clinic Network (DLCN) criteria. **Methods:** This cross-sectional study included 80 patients (mean age, 53.4 [13.2] years; 58% women) receiving maximally tolerated lipid-lowering therapy (statins ± ezetimibe). Serum concentrations of lipoprotein-associated phospholipase A_2_ (Lp-PLA_2_), tumor necrosis factor-α (TNF-α), apolipoprotein B (ApoB), oxidized low-density lipoprotein (oxLDL), and monocyte chemoattractant protein-1 (MCP-1) were measured. Participants were classified as definite FH (DLCN score > 8; n = 45) or non-definite FH (DLCN score ≤ 8; n = 35). Biomarker concentrations were analyzed according to the above subgroups and sex. **Results:** Patients with definite FH had significantly higher serum Lp-PLA_2_ concentrations (62.2 [33.8] vs. 46.5 [19.9] ng/mL; *p* = 0.03) and TNF-α concentrations (6.94 [4.53] vs. 4.51 [2.93] pg/mL; *p* = 0.02) compared to non-definite FH, whereas ApoB, oxLDL, and MCP-1 concentrations did not differ significantly between the subgroups. Women had significantly higher TNF-α concentrations compared to men (6.57 [4.74] vs. 4.60 [2.20] pg/mL; *p* = 0.04) with no differences among other cytokines. **Conclusions:** Patients with definite FH exhibited persistently higher circulating Lp-PLA_2_ and TNF-α concentrations despite maximally tolerated lipid-lowering therapy, suggesting ongoing activation of specific inflammatory pathways. These findings support further investigation of Lp-PLA_2_ and TNF-α as candidate biomarkers for cardiovascular risk assessment in FH.

## 1. Introduction

Familial hypercholesterolemia (FH) is one of the most common inherited metabolic disorders, affecting approximately 1 in 250 individuals worldwide [1,2]. It is characterized by lifelong elevation of low-density lipoprotein cholesterol (LDL-C), resulting in premature atherosclerotic cardiovascular disease (ASCVD). Most genetically confirmed cases are caused by pathogenic variants in LDLR, whereas pathogenic variants in APOB and gain-of-function variants in PCSK9 are less common [3,4,5]. 

The Dutch Lipid Clinic Network (DLCN) criteria are widely used for the clinical diagnosis of FH. They integrate family history, clinical manifestations, physical examination findings, LDL-C concentrations, and, when available, genetic testing. Despite validated diagnostic criteria, FH remains substantially underdiagnosed, and many patients are diagnosed only after developing cardiovascular complications [6].

Although elevated LDL-C is the primary driver of atherosclerosis in FH, cardiovascular risk varies considerably among patients with similar lipid profiles, suggesting that mechanisms beyond LDL-C contribute to disease progression [7,8]. Biomarkers reflecting complementary aspects of lipoprotein-driven atherosclerosis may therefore improve cardiovascular risk assessment beyond conventional lipid measurements. Lp-PLA_2_, TNF-α, oxLDL, MCP-1, and ApoB were selected as they reflect distinct pathophysiological processes, including vascular inflammation, inflammatory activation, oxidative modification of LDL particles, monocyte recruitment, and the burden of atherogenic lipoproteins [9,10,11,12,13]. Unlike hs-CRP and IL-6, which are well-evidenced and established cardiovascular markers and primarily reflect systemic inflammation, these biomarkers provide a more specific assessment of vascular processes directly involved in atherogenesis [14]. 

Evidence linking inflammatory biomarker profiles with the clinical probability of FH defined by the DLCN criteria remains limited. Therefore, this study aimed to evaluate selected biomarkers of inflammation and atherosclerosis in patients with clinically suspected FH stratified according to the DLCN criteria and sex to determine whether these biomarkers provide additional insight into residual inflammatory risk beyond conventional lipid measurements.

## 2. Materials and Methods

### 2.1. Study Population

This cross-sectional study included 80 consecutive adult patients referred to the cardiology outpatient clinic of the Upper Silesian Medical Centre in Katowice (between 2024 and 2025) due to suspected FH. Referral was based on the presence of hypercholesterolemia, a suggestive family or clinical history, and/or physical manifestations consistent with FH.

The inclusion criteria were age ≥18 years and written informed consent. The exclusion criteria were as follows: endocarditis, myocarditis, pericarditis, or systemic vasculitis within the 6 months preceding the study; stroke or transient ischemic attack within the 2 months preceding enrollment; acute or chronic inflammatory conditions, autoimmune diseases, or infectious diseases within the month preceding enrollment; clinically significant acute or chronic kidney disease (estimated glomerular filtration rate [eGFR] < 30 mL/min/1.73 m^2^) or liver disease; thyroid dysfunction (hypothyroidism or hyperthyroidism); malignant neoplasms, lymphoma, or leukemia diagnosed and/or treated within the 2 years preceding enrollment; and a history of alcohol or illicit substance abuse within the year preceding enrollment.

### 2.2. Clinical and Laboratory Assessment

Detailed clinical data were collected for all participants based on medical records and the current clinical evaluation. Cardiovascular risk factors, comorbidities, and previous cardiovascular events were defined according to the European Society of Cardiology (ESC) guidelines [6,15,16,17,18,19].

All patients underwent routine laboratory testing, including fasting serum lipid measurements of total cholesterol (TC), low-density lipoprotein cholesterol (LDL-C), high-density lipoprotein cholesterol (HDL-C), and triglycerides (TG), determined using an enzymatic colorimetric method (Roche Diagnostics GmbH, Mannheim, Germany). Data on ongoing lipid-lowering therapy were obtained from medical records and patient interviews.

### 2.3. FH Diagnosis and Classification

The probability of FH was assessed using the Dutch Lipid Clinic Network (DLCN) criteria [6]. This scoring system incorporates family history, clinical history, physical examination findings, the highest documented serum LDL-C concentration, and results of genetic testing for pathogenic variants in the LDLR, APOB, or PCSK9 genes [20,21]. As a limitation of the present study, systematic genetic screening was not performed. However, results of previously conducted genetic tests were available for 18 patients and were incorporated into the DLCN score when applicable. Among genetically confirmed patients, 14 carried pathogenic LDLR variants (7 women and 7 men) and four carried pathogenic APOB variants (1 woman and 3 men); no pathogenic PCSK9 variants were identified. Accordingly, the study cohort should be regarded as a cohort of patients with clinically suspected FH and partial genetic confirmation rather than a fully genetically confirmed FH cohort. Patients were classified as possible FH (3–5 points), probable FH (6–8 points), or definite clinical FH according to the DLCN criteria (>8 points). For statistical analyses, participants were grouped into the definite clinical FH (DLCN > 8) and the non-definite FH (DLCN ≤8) groups and were further stratified by sex. Throughout the manuscript, findings are interpreted as referring to patients with clinically suspected FH stratified according to the DLCN criteria, rather than to a fully genetically confirmed FH population.

### 2.4. Biomarker Measurements

Fasting blood samples were collected from all participants after at least 3 months of stable, maximally tolerated lipid-lowering therapy consisting of statins and/or ezetimibe. Serum concentrations of biomarkers related to inflammation and atherosclerosis were measured, including Lp-PLA_2_, TNF-α, ApoB, oxLDL, and MCP-1.

All samples were transported under appropriate conditions to the Laboratory of Silesia LabMed Research and Implementation Center at the Medical University of Silesia in Katowice, Poland, where they were stored at −80 °C until further laboratory analyses.

Serum Lp-PLA_2_ levels were measured using a chemiluminescence immunoassay (CLIA) on the MAGLUMI 800 analyzer (Snibe, Shenzhen, China). ApoB concentrations were determined using an immunoturbidimetric method (Snibe Diagnostic, Shenzhen, China). TNF-α and MCP-1 concentrations were quantified using commercially available enzyme-linked immunosorbent assay (ELISA) kits (Cloud-Clone Corp., Katy, TX, USA). OxLDL concentrations were measured using an ELISA kit (FineTest, Wuhan, China). All assays were performed according to the manufacturers’ instructions. All serum samples were analyzed within a single analytical run. According to the manufacturers’ specifications, the intra-assay coefficient of variation was <10% and the inter-assay coefficient of variation was <12% for all ELISAs.

The biomarker panel was predefined to evaluate complementary pathways underlying residual cardiovascular risk in FH rather than systemic inflammatory activity alone; therefore, hs-CRP and IL-6 were not included in the study protocol.

### 2.5. Statistical Analysis

The sample size was calculated based on a comparison of the means of two independent groups. Assuming a significance level (α) of 0.05 and a statistical power (1 − β) of 80% (corresponding to a type II error of 0.20), the minimum total sample size required to detect a medium effect size was estimated to be 52 participants (26 per group).

The distribution of continuous variables was assessed using the Shapiro–Wilk test. Normally distributed data are presented as the mean ± standard deviation (SD) and were compared using Student’s *t*-test. Non-normally distributed data are presented as the median and interquartile range (IQR) and were compared using the Mann–Whitney *U* test. Pearson’s correlation coefficient (*r*) was used to assess the associations between biomarkers and lipid parameters.

All statistical analyses were performed using MedCalc Statistical Software version 23.5 (MedCalc Software Ltd., Ostend, Belgium).

### 2.6. Ethical Approval

The study was conducted in accordance with the principles of the Declaration of Helsinki and was approved by the Bioethics Committee for Scientific Research of the Medical University of Silesia in Katowice, Poland (approval no. PCN/CBN/0022/KB1/77/I/17/21). Written informed consent was obtained from all participants before study enrollment.

This study was supported by a non-commercial research grant from the Medical University of Silesia (PCN-1-178/N/2/Z).

## 3. Results

The study population consisted of 80 patients with a mean age of 53.4 (13.2) years; 47 participants (58%) were women. The baseline clinical characteristics of the study population are summarized in Table 1. Hypertension was present in 51% of patients, whereas obesity and diabetes mellitus were diagnosed in 15% and 6% of patients, respectively. Atherosclerotic cardiovascular disease was common, with coronary artery disease present in 47.5% of patients and peripheral artery disease in 11%. A history of myocardial infarction was reported in 27% of participants, whereas 2.5% had a history of ischemic stroke. Heart failure was diagnosed in 23% of the study population (Table 1).

All participants had hypercholesterolemia and had received maximally tolerated lipid-lowering therapy for at least 3 months before enrollment. Statins were prescribed to 90% of participants, ezetimibe to 70%, and 66% received combination therapy. All participants received at least one lipid-lowering agent (Table 1). Patients were evaluated before being considered for PCSK9 inhibitor therapy. Despite intensive lipid-lowering treatment, mean lipid concentrations remained elevated: total cholesterol, 258.3 (86.1) mg/dL; LDL-C, 177 (75) mg/dL; and triglycerides, 130 (61) mg/dL (Table 2).

Lipid profile values presented in Table 2 represent laboratory measurements obtained during qualification for the PCSK9 inhibitor reimbursement program and reflect persistent hypercholesterolemia despite maximal tolerated lipid-lowering therapy.

According to the DLCN criteria, 45 patients (56%) were classified as having definite FH. The remaining participants were classified as having probable FH (9%), possible FH (11%), or unlikely FH (24%) and were combined into the non-definite FH group for subsequent analyses. The most frequent contributors to the DLCN score were clinical history (28 patients), family history (21 patients), physical examination findings (13 patients), and LDL-C levels (17 patients). Positive genetic test results confirming pathogenic variants in the *LDLR* or *APOB* genes were available for 18 patients and were incorporated into the DLCN score when applicable. However, systematic genetic screening was not performed as part of the study (Table 2).

Previous genetic testing results were available for 18 participants (22.5%). Fourteen patients carried pathogenic LDLR variants (7 women and 7 men), whereas four patients carried pathogenic APOB variants (1 woman and 3 men). No pathogenic PCSK9 variants were identified. Because of the limited number of genetically confirmed cases, formal subgroup analyses were not performed.

Patients with definite FH were significantly younger than those with non-definite FH (49.8 [13.0] vs. 58.0 [11.5] years; *p* = 0.005). They also had significantly higher maximum documented LDL-C concentrations (315 [87] vs. 185 [67] mg/dL; *p* < 0.001).

### Serum Biomarkers of Atherosclerosis and Inflammation

**Figure 1:**
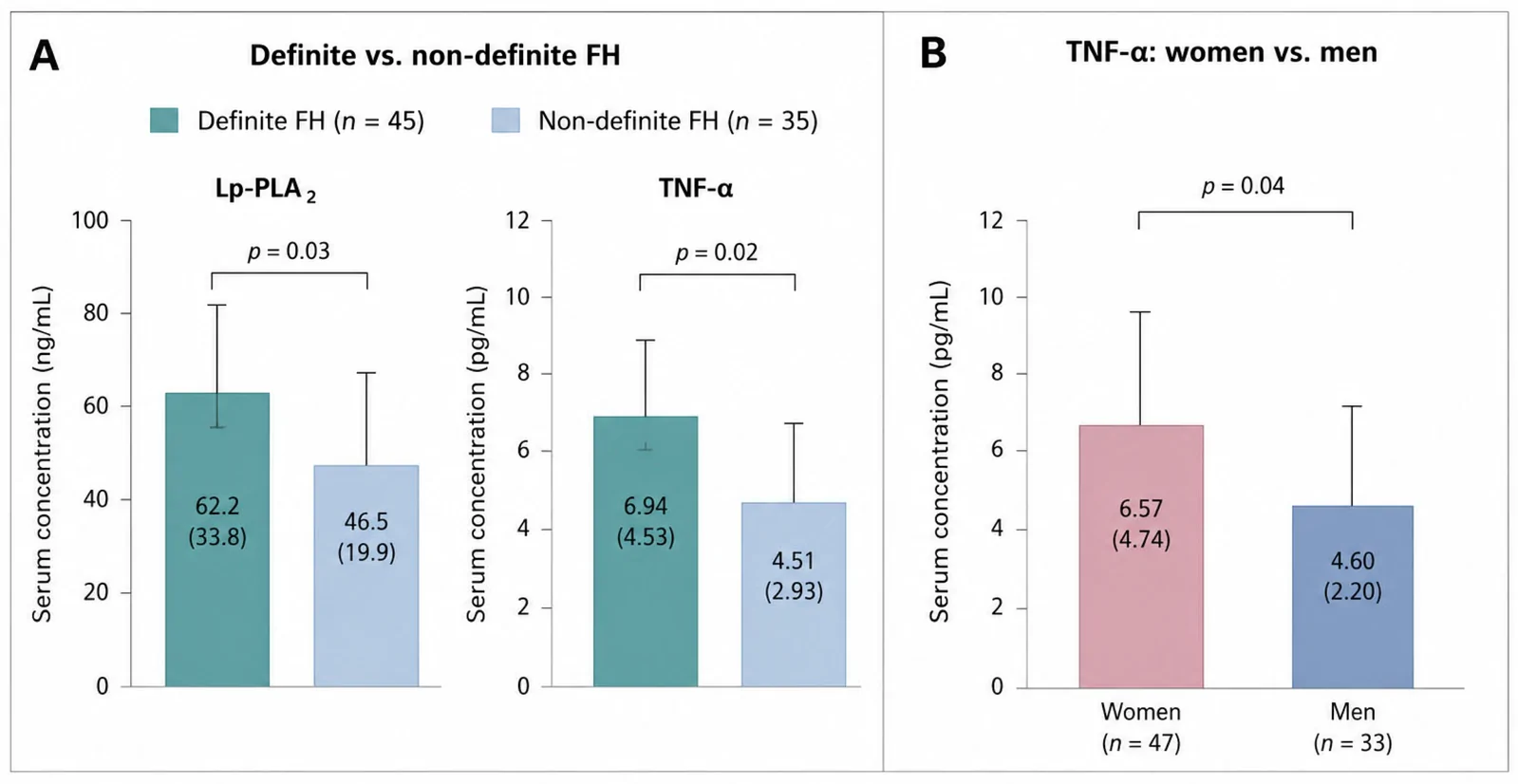
Serum concentrations of selected biomarkers according to FH status and sex. (**A**) Mean (SD) serum concentrations of lipoprotein-associated phospholipase A_2_ (Lp-PLS_2_) and tumor necrosis factor-α (TNF-α) in patients with definite FH (DLCN score > 8) and non-definite FH (DLCN score ≤ 8). (**B**) Mean (SD) serum concentrations of TNF-α in women and men. FH—familial hypercholesterolemia; DLCN—Dutch Lipid Clinic Network; SD—standard deviation.

Mean serum Lp-PLA_2_ concentrations were significantly higher in patients with definite FH than in those with non-definite FH (62.2 [33.8] vs. 46.5 [19.9] ng/mL; *p* < 0.05; Figure 1A). Similarly, serum TNF-α concentrations were significantly higher in the definite FH group (6.94 [4.53] vs. 4.51 [2.93] pg/mL; *p* < 0.05; Figure 1A). In contrast, no significant between-group differences were observed in serum ApoB, oxLDL, or MCP-1 concentrations. Mean ApoB concentrations were higher in patients with definite FH than in those with non-definite FH (0.88 [0.37] vs. 0.77 [0.31] g/L), although the difference was not statistically significant (*p* = 0.14). Serum MCP-1 concentrations also tended to be higher in the definite FH group (198.0 [110.1] vs. 173.2 [70.3] pg/mL), but this difference did not reach statistical significance (*p* = 0.24). Likewise, serum oxLDL concentrations were comparable between the two groups (455.8 [217.2] vs. 446.6 [174.2] ng/mL; *p* = 0.84).

When serum biomarker concentrations were analyzed according to sex, regardless of DLCN category, TNF-α was the only marker that differed significantly between women and men. Female patients had significantly higher TNF-α concentrations than male patients (6.57 [4.75] vs. 4.60 [2.20] pg/mL; *p* < 0.05; Figure 1B). No sex-related differences were observed for Lp-PLA_2_, ApoB, oxLDL, or MCP-1.

Furthermore, oxLDL showed a moderate positive association with MCP-1 (*r* = 0.45, *p* < 0.01), whereas ApoB concentrations were strongly associated with Lp-PLA_2_ concentrations (r = 0.60, *p* < 0.01). No other significant correlations were identified among the assessed biomarkers. None of the biomarkers was significantly associated with on-treatment LDL-C concentrations.

## 4. Discussion

This study provides a comprehensive assessment of inflammatory and atherosclerosis-related biomarkers in patients with clinically suspected FH stratified according to the DLCN criteria. Unlike previous studies that focused on individual biomarkers or genetically confirmed FH, our study evaluated a panel of complementary biomarkers reflecting distinct pathophysiological mechanisms in a real-world population receiving maximally tolerated lipid-lowering therapy. This approach enabled the assessment of residual inflammatory risk beyond LDL-C reduction.

A key finding of the present study is the persistent elevation of Lp-PLA_2_ and TNF-α concentrations in patients with definite FH despite maximally tolerated lipid-lowering therapy, whereas the remaining biomarkers were comparable between the study groups. These findings suggest selective activation of inflammatory pathways that are not captured by conventional lipid measurements and appear to be independent of on-treatment LDL-C concentrations, supporting the potential role of these biomarkers in cardiovascular risk stratification. Moreover, oxLDL was moderately correlated with MCP-1, whereas ApoB showed a strong positive association with Lp-PLA_2_. The study population was homogeneous with respect to lipid-lowering treatment, as all patients received maximally tolerated therapy. Nevertheless, differences in biomarker concentrations remained evident across groups stratified according to the DLCN criteria. Notably, none of the assessed biomarkers was associated with on-treatment LDL-C concentrations, suggesting that these markers may provide information beyond conventional lipid parameters. Additionally, no sex-related differences were observed for any biomarkers, except for TNF-α, which was significantly higher in women than in men.

To our knowledge, this is the first study to compare serum TNF-α, MCP-1, oxLDL, and ApoB concentrations between patients with definite and non-definite FH. Furthermore, our findings demonstrated significantly higher TNF-α concentrations in patients with definite FH, with women exhibiting higher levels compared to men.

These findings support our hypothesis that inflammatory pathways may contribute to the more advanced clinical phenotype of FH and may be associated with increased atherosclerotic risk.

### 4.1. Lp-PLA_2_

Our findings regarding Lp-PLA_2_ are consistent with previous reports. Mattina et al. demonstrated significantly higher Lp-PLA_2_ activity in patients with definite FH than in those with non-definite FH, which was independent of LDL-C concentrations and statin treatment [22]. Elevated Lp-PLA_2_ may reflect enhanced arterial inflammation, which was proposed as a marker associated with the markedly increased cardiovascular risk observed in FH, partly because of the predominance of small, dense LDL particles in hypercholesterolemic states. Mechanistically, Lp-PLA_2_ catalyzes the hydrolysis of oxidized phospholipids, generating pro-inflammatory and pro-atherogenic mediators that promote endothelial dysfunction and atherogenesis [23,24].

### 4.2. TNF-α

In the present study, patients with definite FH exhibited significantly higher serum TNF-α concentrations than those with non-definite FH. These findings are consistent with those reported by Holven et al., who demonstrated increased expression of TNF-related genes in patients with FH receiving long-term statin treatment [25]. In contrast, PCSK9 inhibitors have been shown to reduce TNF-α concentrations in statin-resistant patients with FH [26].

Notably, in our cohort, persistent TNF-α elevation despite maximally tolerated lipid-lowering therapy was more pronounced in women than in men. This observation suggests sex-specific differences in the inflammatory profile of patients with FH and indicates that the interactions between lipid metabolism and inflammation may be more complex than previously recognized.

Experimental studies indicate that estrogen downregulates NF-κB signaling and cytokine production, which has been proposed to explain the lower TNF-α concentrations typically observed in women in other clinical settings [27]. However, this mechanism does not explain the elevated TNF-α concentrations observed in women in the present study. Holven and Roeters van Lennep reported that sex-related differences in lipid profiles are more pronounced in FH than in the general population. Women with FH generally exhibit lower LDL-C and higher HDL-C concentrations during early adulthood and midlife, whereas female-specific physiological transitions, such as pregnancy and menopause, substantially influence lipid metabolism and cardiovascular risk. Taken together, these findings are in contrast with the pro-inflammatory profile observed in our cohort and may be related to hormonal status as well as metabolic and inflammatory differences in women with FH [28].

TNF-α contributes to atherogenesis through multiple mechanisms, including endothelial activation, upregulation of adhesion molecules, promotion of monocyte recruitment, and enhancement of oxidative stress, collectively facilitating foam cell formation. These findings further support the concept that the pathophysiology of FH extends beyond lipid accumulation and suggest that persistent inflammation may contribute to ongoing atherosclerotic progression despite standard lipid-lowering therapy [29].

In contrast to Lp-PLA_2_ and TNF-α, no significant differences were observed in the remaining biomarkers between patients with definite and non-definite FH. Nevertheless, a trend toward higher ApoB concentrations was observed in patients with definite FH. Tremblay et al. demonstrated substantial metabolic alterations in FH, including markedly increased very-low-density lipoprotein (VLDL) ApoB100 production, enlarged LDL ApoB pools, and reduced LDL catabolism [30]. These abnormalities reflect increased hepatic ApoB synthesis together with impaired clearance of ApoB-containing lipoproteins. Consequently, elevated ApoB concentrations indicate a greater number of circulating atherogenic lipoprotein particles, thereby promoting their retention within the arterial wall and increasing the overall atherogenic burden [31].

Serum MCP-1 concentrations were comparable between the study groups, which may be attributable to the widespread use of intensive lipid-lowering therapy. Such treatment may attenuate oxidative stress and monocyte activation [32]. Morales-Portano et al. demonstrated that PCSK9 inhibition significantly reduced both LDL-C and MCP-1 concentrations in statin-resistant patients with FH [26]. Reduced MCP-1 expression limits monocyte recruitment to the vascular wall, thereby contributing to plaque stabilization and slowing the progression of atherosclerosis [33].

OxLDL concentrations did not differ significantly between the DLCN categories. Previous studies have demonstrated persistently elevated oxLDL concentrations despite substantial reductions in LDL-C achieved with statin therapy [34]. These findings indicate the need for adjunctive therapeutic strategies. Persistent oxidative stress, chronic inflammation, and endothelial dysfunction may sustain LDL oxidation independently of circulating LDL-C concentrations, highlighting the potential value of therapeutic approaches that target mechanisms beyond conventional lipid lowering [35].

### 4.3. Study Limitations

This cross-sectional study was conducted in a relatively small cohort, and systematic genetic testing was not performed, precluding confirmation of FH-associated pathogenic variants in all participants. Moreover, only 18 of the 80 enrolled patients had previous molecular confirmation of familial hypercholesterolemia. Therefore, our findings should be interpreted as applicable to a population with clinically suspected FH classified according to the Dutch Lipid Clinic Network (DLCN) criteria, rather than to genetically confirmed FH. Nevertheless, the study population was homogeneous with respect to severe hypercholesterolemia and treatment with maximally tolerated lipid-lowering therapy, reflecting routine clinical practice.

Additional limitations include the single-center design, the lack of longitudinal follow-up, and the absence of long-term cardiovascular outcome assessment, which precluded evaluation of the prognostic value of the investigated biomarkers.

Lipid-lowering therapy represents an important potential confounder in the assessment of inflammatory and atherosclerosis-related biomarkers. All participants had received maximally tolerated lipid-lowering therapy for at least 3 months before enrollment, most commonly statins administered either as monotherapy or in combination with ezetimibe. Given the well-established pleiotropic anti-inflammatory effects of statins, including reductions in pro-inflammatory cytokines such as TNF-α and modulation of Lp-PLA_2_ activity, the measured biomarker concentrations may underestimate the true inflammatory burden associated with FH and attenuate differences between the study groups. Although this reflects contemporary clinical practice, it limits the ability to distinguish disease-specific effects from treatment-related effects.

It should also be noted that the lipid profile presented in Table 2 corresponds to measurements obtained during qualification for the PCSK9 inhibitor reimbursement program, whereas inflammatory biomarkers were assessed after at least 3 months of stable maximally tolerated lipid-lowering therapy, individualized according to treatment limitations such as statin intolerance or reimbursement restrictions for PCSK9 inhibitors. Consequently, the historical maximum LDL-C concentration, the lipid profile obtained during qualification for PCSK9 inhibitor therapy, and the on-treatment LDL-C concentrations represent distinct clinical time points. Despite maximally tolerated lipid-lowering therapy, many patients remained above guideline-recommended LDL-C targets because PCSK9 inhibitor therapy had not yet been initiated.

Circulating inflammatory biomarkers are also influenced by coexisting inflammatory conditions. To minimize this potential source of bias, patients with known autoimmune diseases were excluded, and blood sampling was postponed during acute infections. Nevertheless, subclinical or unrecognized inflammatory processes cannot be excluded and may have contributed to residual variability. In addition, patient-related factors such as body mass index, smoking status, and hormonal status in women, particularly menopausal status, were not systematically controlled for despite their recognized influence on inflammatory pathways. This is particularly relevant in light of the observed sex-related differences in TNF-α concentrations. Future studies should incorporate these variables into multivariable models or stratified analyses to better define the independent contribution of FH to systemic inflammation.

Another important limitation is the cross-sectional design of the study, which precludes causal inference and assessment of temporal relationships. In addition, no longitudinal follow-up or cardiovascular outcome data were available; therefore, the prognostic significance of the investigated biomarkers and their incremental predictive value beyond LDL-C levels or the DLCN score could not be determined. Prospective studies with long-term follow-up and hard cardiovascular endpoints are warranted to validate these findings.

### 4.4. Clinical Implications

The present findings have several important clinical implications. Assessment of serum Lp-PLA_2_ and TNF-α concentrations may improve cardiovascular risk stratification in patients with FH and help identify individuals who may benefit from intensified preventive strategies. Persistent inflammatory activation despite maximally tolerated lipid-lowering therapy suggests that additional therapeutic approaches beyond statins and ezetimibe may be warranted [36].

A personalized, patient-centered approach incorporating FH-specific inflammatory biomarkers may improve risk assessment and facilitate more individualized management in this high-risk population. Our findings further highlight the marked heterogeneity of cardiovascular risk among patients with FH, indicating that some individuals may require treatment intensification beyond conventional lipid-lowering therapy.

Future research should focus on large prospective studies evaluating both the prognostic value of these biomarkers and the anti-inflammatory effects of emerging therapeutic strategies. Integration of inflammatory biomarkers with the DLCN classification may further refine cardiovascular risk assessment in patients with suspected FH [37].

## 5. Conclusions

Patients with definite familial hypercholesterolemia exhibited significantly higher circulating Lp-PLA_2_ and TNF-α concentrations than those with non-definite FH despite maximally tolerated lipid-lowering therapy, suggesting persistent activation of specific inflammatory pathways. In contrast, ApoB, oxLDL, and MCP-1 concentrations did not differ significantly between the study subgroups. The higher TNF-α concentrations observed in women may indicate sex-related differences in inflammatory responses that warrant further investigation.

Given the cross-sectional design and the absence of longitudinal cardiovascular outcome data, these findings should be considered hypothesis-generating. Lp-PLA_2_ and TNF-α represent promising candidate biomarkers for future evaluation in patients with FH. However, their incremental prognostic value beyond established lipid parameters and the DLCN classification requires confirmation in larger prospective studies with long-term follow-up and hard cardiovascular endpoints.

## Figures and Tables

**Table 1:** Baseline demographic and clinical characteristics of the study population (n = 80).

Characteristic	Value
**Demographic characteristics**	
Age, years	53.4 ± 13.2
Female sex, n (%)	47 (58%)
BMI, kg/m^2^	27.4
Obesity, n (%)	12 (15%)
**Medical history**	
Hypertension, n (%)	41 (51%)
Grade I	32 (40.0%)
Grade II	9 (11.3%)
Grade III	0
Diabetes mellitus, n (%)	5 (6%)
Coronary artery disease, n (%)	38 (47.5%)
One-vessel disease	7 (8.7%)
Two-vessel disease	12 (15.0%)
Three-vessel disease	19 (23.7%)
Previous myocardial infarction, n (%)	22 (27.5%)
Coronary artery bypass grafting, n (%)	12 (15%)
Heart failure, n (%)	14 (17.5%)
HFpEF	10 (12.5%)
HFmrEF	3 (3.7%)
HFrEF	1 (1.2%)
Previous ischemic stroke, n (%)	2 (2.5%)
Peripheral artery disease, n (%)	9 (11.2%)
**Current lipid-lowering therapy**	
Statins, n (%)	72 (90%)
Rosuvastatin	56 (70%)
Atorvastatin	16 (20%)
Ezetimibe, n (%)	56 (70%)
Combination therapy, n (%)	53 (66%)
**Other cardiovascular medication**	
Acetylsalicylic acid (ASA), n (%)	52 (65%)
β-blockers, n (%)	65 (81.2%)
ACE inhibitors/ARBs, n (%)	45 (56.2%)
Diuretics, n (%)	20 (2%) *

Abbreviations: BMI, body mass index; HFpEF, heart failure with preserved ejection fraction; HFmrEF, heart failure with mildly reduced ejection fraction; HFrEF, heart failure with reduced ejection fraction; ACE, angiotensin-converting enzyme; ARB, angiotensin receptor blocker. * Number of patients (percentage).

**Table 2:** Lipid profile and Dutch Lipid Clinic Network assessment.

**A. Lipid Profile**		
**Lipid Parameter**	**Mean ± SD**	**P25–P75**
Total cholesterol, mg/dL	258.3 ± 86.1	211–297
HDL-C, mg/dL	56 ± 19	43–66
LDL-C, mg/dL	177 ± 75	123–212
Triglycerides, mg/dL	130 ± 61	86–168
**B. Dutch Lipid Clinic Network criteria**		
**Criterion**	**Score/category**	**Patients, n**
Family history	0 points	35
	1 point	24
	2 points	21
Clinical history	0 points	46
	1 point	6
	2 points	28
Physical examination	0 points	62
	4 points	5
	6 points	13
LDL cholesterol	1 point	19
	3 points	19
	5 points	22
	8 points	17
Genetic mutation	LDLR	14
	APOB	4
	PCSK9	0
**C. FH diagnosis according to DLCN score**		
**FH classification**	**DLCN score**	**Patients, n**
Definite FH	>8 points	45
Probable FH	6–8 points	7
Possible FH	3–5 points	9
Unlikely FH	<3 points	19

Data are presented as mean ± standard deviation (SD), interquartile range (P25–P75), or number of patients, as appropriate. Abbreviations: APOB, apolipoprotein B; DLCN, Dutch Lipid Clinic Network; FH, familial hypercholesterolemia; HDL-C, high-density lipoprotein cholesterol; LDL-C, low-density lipoprotein cholesterol; LDLR, low-density lipoprotein receptor; PCSK9, proprotein convertase subtilisin/kexin type 9.

## Data Availability

The data presented in this study are available on request from the corresponding author.
